# Pilot of a Priorities Aligned Decision-Making Microskills Curriculum

**DOI:** 10.1093/geroni/igaf122.2277

**Published:** 2025-12-31

**Authors:** Jennifer Oullet, Eliza Kiwak, Loni Belyea, Brenda Nettles, Mallory Brown, Mary Tinetti, Timothy Farrell, Natalie Sanders

**Affiliations:** Yale School of Medicine, New Haven, Connecticut, United States; Yale School of Medicine, New Haven, Connecticut, United States; Hello Alpha, Southern Pines, North Carolina, United States; Johns Hopkins School of Nursing, Baltimore, Maryland, United States; The University of North Carolina at Chapel Hill, Chapel Hill, North Carolina, United States; Yale University, New Haven, Connecticut, United States; University of Utah, Spencer Fox Eccles School of Medicine, Salt Lake City, Utah, United States; University of Utah, Spencer Fox Eccles School of Medicine, Salt Lake City, Utah, United States

## Abstract

Patient Priorities Care (PPC) provides an approach for identifying patient health care priorities and aligning care with those priorities. Training in PPC has demonstrated high overall satisfaction and its implementation is associated with positive patient centered outcomes. Yet, the current training approach is time and resource intensive. This work describes the adaptation and piloting of a PPC curriculum to address these barriers. An interdisciplinary group of clinician educators collaborated to divide PPC content into 8 core skills and organize it into graphic power point and PDF documents. Educators are piloting materials with learners. A baseline survey was administered to pilot participants (educators and learners). Participants will complete a post-pilot survey to assess feasibility and ease of use of materials. Eighteen learners have completed the baseline survey. Most learners were from either Internal Medicine (22%) or Geriatric Medicine (61.1%) disciplines. More than half (55.6%) had some prior training in priorities-aligned care. On a scale of 1-5 (1 not confident and 5 extremely confident), learners reported being moderately confident (range 22.2%-44.4%), quite confident (range 27.8%-44.4%) or extremely confident (range 11.1%-22.2%) in the 8 core skills. A high level of baseline confidence in the core skills will allow assessment of content feasibility. Next steps include evaluating ease of use of materials and refining the materials and assessment methods based on feedback. We will then disseminate curricular materials and assessment methods to educators nationally. We hope to demonstrate that the adapted curriculum will ensure durable implementation of PPC across multiple health care settings.

